# Ontological how and why: action and objective of planned processes in the food domain

**DOI:** 10.3389/frai.2023.1137961

**Published:** 2023-07-04

**Authors:** Damion Dooley, Tarini Naravane

**Affiliations:** ^1^Centre for Infectious Disease Genomics and One Health, Simon Fraser University, Burnaby, BC, Canada; ^2^Biological Systems Engineering, University of California, Davis, Davis, CA, United States

**Keywords:** food processing, ontology, mechanism, action, data specification, material processing

## Abstract

The computational modeling of food processing, aimed at various applications including industrial automation, robotics, food safety, preservation, energy conservation, and recipe nutrition estimation, has been ongoing for decades within food science research labs, industry, and regulatory agencies. The datasets from this prior work have the potential to advance the field of data-driven modeling if they can be harmonized, but this requires a standardized language as a starting point. Our primary goal is to explore two interdependent aspects of this language: the granularity of process modeling sub-parts and parameter details and the substitution of compatible inputs and processes. A delicate semantic distinction—categorizing planned processes based on the objectives they seek to fulfill vs. categorizing them by the actions or mechanisms they utilize—helps organize and facilitate this endeavor. To bring an ontological lens to process modeling, we employ the Open Biological and Biomedical Ontology Foundry ontological framework to organize two main classes of the FoodOn upper-level material processing hierarchy according to objective and mechanism, respectively. We include examples of material processing by mechanism, ranging from abstract ones such as “application of energy” down to specific classes such as “heating by microwave.” Similarly, material processing by objective—often a transformation to bring about materials with certain qualities or composition—can, for example, range from “material processing by heating threshold” to “steaming rice”.

## 1. Introduction

The post-harvest treatment of food up to the point of consumption from both industrial and domestic food preparation perspectives is an active area of research that is not yet comprehensively covered by an integrated set of ontologies. Here, we propose for discussion, as part of a larger life-sciences family of ontologies, the basic terms required for a standardized process ontology that can enable and integrate data-driven analysis of research datasets on the one hand and (with data at the relevant resolution and data size) support dynamic process control applications on the other hand. Formalized language is required to integrate what have often been siloed food composition datasets (FCD) containing foods that result from simple processes such as boiling, freezing, and roasting. Standardized language is a prerequisite to the manual or automated alignment of different food entities across FCD datasets. For example, the nutritional content of frozen carrots can only be compared across datasets if the experimental protocols for storing, soaking, blanching, boiling, and (flash) freezing processes are comparable (Hinojosa-Nogueira et al., 2021; Westenbrink et al., 2021).

Ontologies can be used to normalize the comparable portions of data selected from the body of scientific literature on food processing so that data-driven analysis and models can be used to address a range of research questions/hypotheses on the causal factor(s) driving sensory and nutritive effects.

This process ontology work also aims to support dynamic process control by providing a framework for describing process input and output phenotype objective thresholds that can trigger mechanism start/stop/pause operations and by providing a framework for choosing among comparable mechanisms to achieve an objective. The ontology has been designed to differentiate between the objectives and mechanisms of a process and to address food processing at both macro (food product) and molecular scale transformations. The latter is especially challenging to describe in food science literature datasets (a task similar to material science engineering modeling). We provide a macro/micro transformation example model that shows the parallels between the food entity and molecular resolutions. This framework addresses the various details of a process to support various decision-making methods for dynamic process control, ranging from simple inferences/linear relationships to more complex ML models.

Given the natural context of food as mainly derived from organisms, our process ontology leverages the framework established by the Open Biological and Biomedical Ontology Foundry (OBO) consortium of ontologies (Jackson et al., 2021), which focuses on life science research. In this study, the modeling of natural physio-chemical or biologically rooted processes can be found in places such as the Gene Ontology's cellular metabolic process [GO:0008152] branch classes (including, for example, fermentation, an enzyme-catalyzed process), which are triggered when some combination of materials and/or environmental context aligns. Unplanned processes can be controlled by planned processes that exhibit human or computer agency/intentionality. To organize processes that satisfy various objectives in transforming things, OBO's Ontology for Biomedical Investigations (OBI) (Bandrowski et al., 2016) introduced the “planned process” class [OBI:0000011], which contains processes that execute a “plan specification” and include a set of instructions and/or objectives.

A recent paper (Dooley et al., 2022) covers a gap analysis of the technical side of modeling processes using W3C OWL ontologies (SOSA, SSN, PO2, and OWL-Time) in comparison to an OBO Foundry ontology approach and recommendations, implemented mainly in OBO Foundry's FoodOn food ontology (Dooley et al., 2018) for extending OBO with some select relationships and classes adapted from the aforementioned ontologies to fill the gap. The paper details the OBI “planned process” related classes and relations and discusses how experimental independent and dependent variables, observations, and characteristics of materials could be structured in a multi-step process model. A brief discussion of measurement data properties is included, but in that (and current) work, we avoid focusing on this topic and note that upcoming OBO work will recommend knowledge graph data structures for measurement values. That paper finishes with a simple recipe model that illustrates ingredient input and output relations at work in a sample selection of food processes but skirts the issue of organizing a hierarchy of food processes; our new work focuses on this topic.

A plan specification can have one or more “action specifications” [IAO:0000007] parts that directly or indirectly control the input material's environmental parameters, such as container pressure, kinetic or thermal energy exposure, or the addition of chemicals or biological substances. An action specification might be to operate a tool or device setting or control to some effect or to give hands-on instruction to an operator to shape a material directly or combine materials. It may reference other planned processes or directly control (via duration, catalysts, or energy supply) an unplanned physio-chemical process. Natural fermentation would be considered unplanned, but a planned process can harness it through action specification(s), devices, and subprocess stages. Other examples are the application of force to a material or to a blade in the material; introducing bacteria to a food substance; controlling atmospheric storage conditions for food; or allowing the fruit to ripen before harvest or consumption (Osorio et al., 2013).

In time, the effect of environmental interventions (whether constant or in flux) yields physical or chemical changes in material input(s) that satisfy process objectives. A material processing “objective specification” is often an expression of the quality(ies) or phenotype(s) of the output material, such as “water at 100 degrees Celsius”, which is the causal result of the process. Other examples are sensory, logistical, food safety, or food formulation functional objectives. In short, an objective is an expression of some desired state of affairs, and the “Process by Objective” class, thus, necessarily includes such an expression either as a final output specification to reach or by a formula of operating parameters. The recognition that an objective has been attained (whether by a human or a device) can be a component objective of a larger process.

## 2. Methodology and results

### 2.1. Process terms

In OBO, currently, there are no “convenience classes” for organizing processes by action or objective, so our proposal involves adding those new process terms and underlying ones within an appropriate OBO ontology. FoodOn could temporarily accommodate them, but OBO's best practice entails consulting about the possible adoption of mid/upper-level terms by the curators of OBI or the in-development Core Ontology for Biology and Biomedicine (COB) (Core Ontology for Biology and Biomedicine, n.d.), which is taking on commonly used OBO terms. Although specific processes such as boiling are examined here in the food context, they are also often applicable to other domains such as manufacturing and laboratory procedures and are best curated in a general-purpose ontology from which FoodOn can draw. Note that in this study, the “is-a” relationship in the legend refers to OWL rdfs:subclassOf. Additionally, all illustrated relations are RO or OBI ontology relations and have their domain and range constraints held in those ontologies (such as RO “has quality” range “quality” [PATO:0000001]).

**Figure 1:**
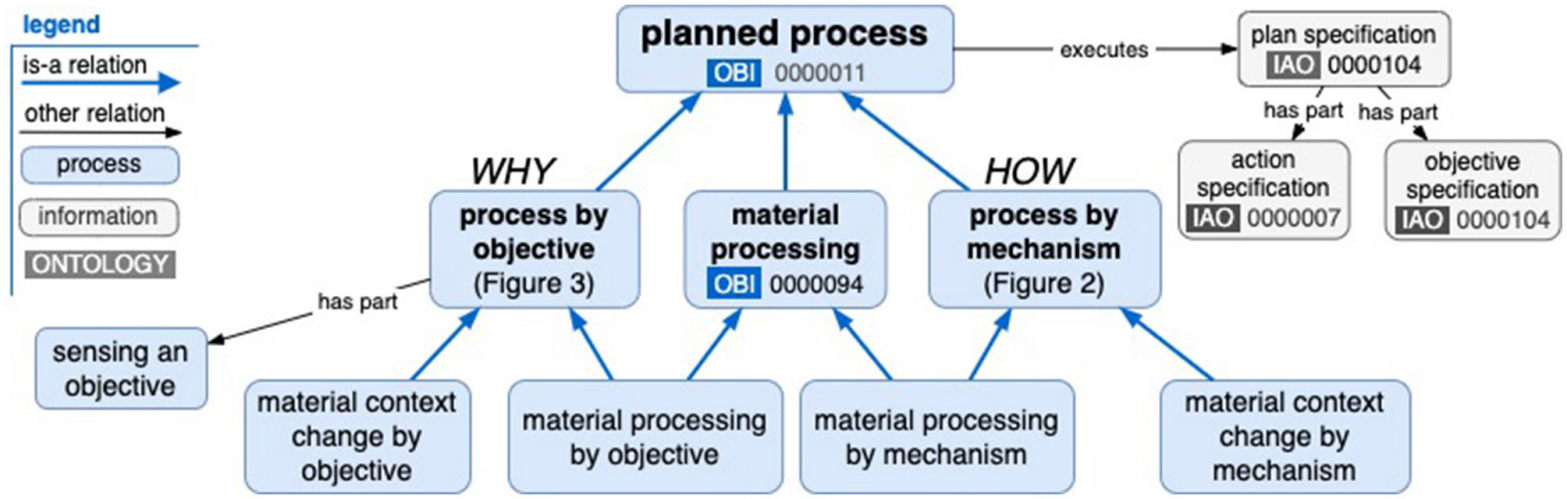
A new material processing hierarchy organized under a planned process, with new “process by objective” and “process by mechanism” branches.

FoodOn has an existing “food transformation process” branch, which will be reorganized according to the scheme proposed below. The branch is managed according to a common OBO term maintenance pattern as a spreadsheet template (FoodOn Robot Tables, 2023), which is periodically converted into a stand-alone ontology import file. Figure 1 offers an overview of the new proposed hierarchy with new “process by objective” and “process by mechanism” classes alongside the existing OBI “material processing” term. The “material context change by objective” and “material context change by mechanism” classes cover both packaging and moving of material entities (to some objective location or by some mechanism of transport), but they are not detailed here.

**Figure 2:**
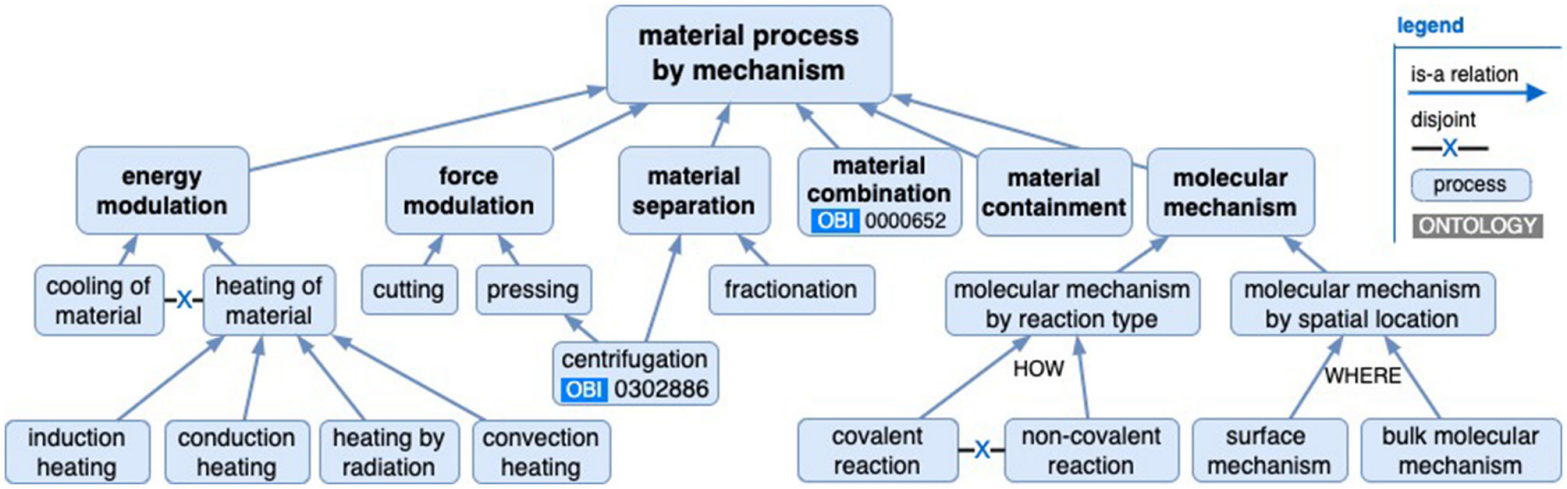
A material processing by mechanism hierarchy with example subclasses, in which no absolute end-point material qualities are specified.

The “material processing by mechanism” branch outlined in Figure 2 covers the application of force or energy and combining materials (and includes some example subclasses). In this study, the relative change effected by a process will modulate a material quality, such as by reducing particle size, changing temperature, or adding a new quality, but it will not specify an absolute threshold upon which to complete the process. These processes continue unless some inherent process limit occurs, such as an exhausted resource or, with mixing miscible liquids, if maximum homogenization is reached.

**Figure 3:**
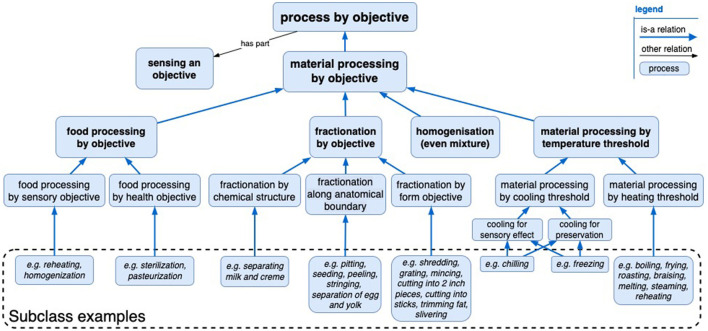
A hierarchy of “material processing by objective” classes and some lower-level examples. Each has a plan specification containing an objective specification that supplies a completion metric.

The “material processing by objective” branch outlined in Figure 3 includes complete processes when one or more objectives are satisfied. This can involve objectives that are expressed as threshold qualities of a material, such as a turkey with a core temperature of 70°C. Alternatively, objectives may be expressed as characteristics of the process—for example, its duration, energy, or amount of catalyst consumed—which are a proxy for predicted material outcomes. When applied to food products, terms such as “chilling” may have highly industry-specific objective semantics, such as the chilling of animal products (Temperatures and Chilling and Freezing Procedures, 2023), which could be formalized in the ontology. The proposed material processing by objective hierarchy does not preclude objective specifications, so a reasoner should be able to infer that material processing by objective classes falls under more general process mechanism classes, for example, “material processing by cooling threshold” as a subclass of “cooling of the material,” or “fractionation by objective” as a subclass of “fractionation”.

**Figure 4:**
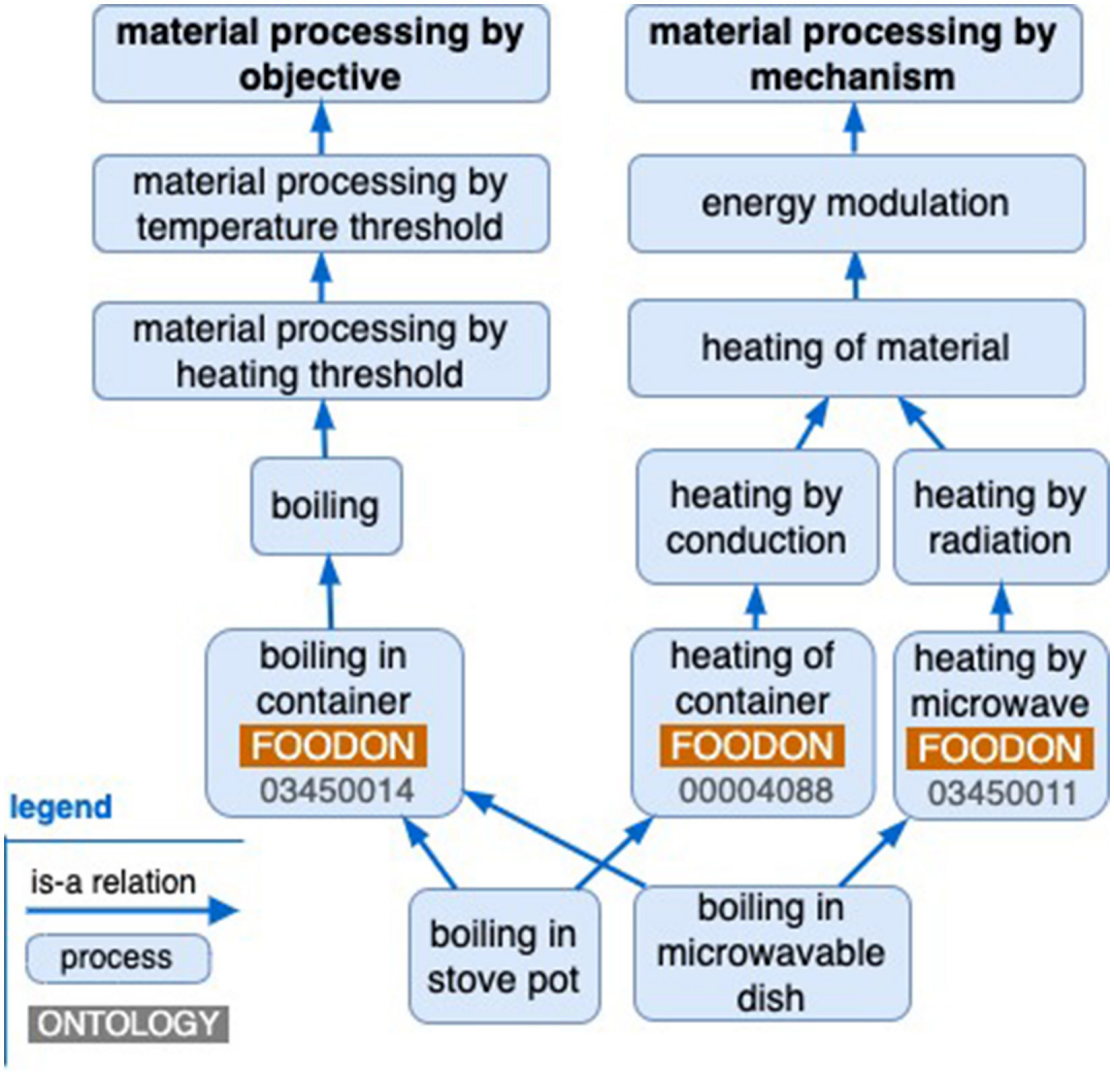
A sketch of polyhierarchical process dependencies.

Positioning of a process by objective (for example, bringing a liquid to its boiling point) and by mechanism (for example, “heating by microwave” or “direct heating of container”) is shown in Figure 4, as is the example of the polyhierarchy of stove top and microwave boiling processes.

While some terms mentioned in the above figures (shown with identifiers) come from existing OBO Foundry and other ontologies, the bulk of this upper-level hierarchy must be created. New key terms are listed in Table 1, along with their definitions and notes. Our motivation for presenting these terms here is to encourage feedback in the spirit of an open-source community so that their labels and definitions can be finalized. Discussion can be held at the GitHub FoodOn issue page https://github.com/FoodOntology/foodon/issues/262 or by contacting the authors directly.

**Table 1:** A new process hierarchy based on objective and mechanism branches.

**Label**	**Definition**	**Notes**
Planned process (OBI)	A process that realizes a plan that is the concretization of a plan specification.	This term is from OBI. Paraphrasing: a process that executes a plan specification.
Process by mechanism	A planned process that has one or more action specification parts in its plan specification that control a mechanism.	A convenience class for organizing processes by their physical mechanism or digital algorithm. An action may be physical, such as pushing a button or setting a dial, or it may be about running some software.
Material processing (OBI)	A planned process that results in physical changes in a specified input material.	More than one input material may be involved. Note that ENVO's similar “material transformation process” is unplanned.
Material processing by mechanism	Material processing has one or more action specifications in its plan specification.	This should also be inferred under “process by mechanism.” Here, action specifications directly or indirectly control a material input's environmental parameters.
Energy modulation	A material processing mechanism in which energy is removed from or added to a material entity.	
Heating of material	An energy modulation in which thermal energy (heat) is applied to a material or its environment.	
Cooling of material	An energy modulation in which thermal energy (heat) is removed from a material or its environment.	
Force modulation	A material processing mechanism in which force is applied to a material or its environment.	
Separating material	A material processing mechanism in which materials are separated.	
Combining material	A material processing mechanism in which materials are combined.	
Molecular mechanism	Material processing is described for specific molecules in the material.	
The molecular mechanism by reaction type	A molecular mechanism is categorized by its reaction type.	
Covalent reaction	A molecular mechanism by reaction type involving a covalent reaction.	
Non-covalent reaction	A molecular mechanism by reaction type involving a non-covalent reaction.	
The molecular mechanism by spatial location	A molecular mechanism is categorized by the region in which reactions occur.	
Surface mechanism	A molecular mechanism where reactions occur at some surface boundary.	
Bulk molecular mechanism	A molecular mechanism where reactions occur throughout a mixture.	
Process by objective	A planned process that has one or more objective specification parts in its plan specification.	A convenience class under which various processes can be grouped or inferred by their objectives.
Material processing by objective	Material processing that has one or more objective specification parts in its plan specification.	This will also be inferred under “process by objective.” Here, processes having equivalent objectives can be swapped.
Direct heating of container (FoodOn)	A heating container process in which the container conducts heat by being near an open flame, a hot surface, or an oven.	
Boiling	A material processing by the heating threshold in which the objective is to keep a liquid at its boiling temperature under atmospheric conditions.	
Material context change	A planned process in which the relation of the input material entity and its proximate environment changes.	
Material context change by objective	A material context change in which the objective is to change the contextual relation of the input material entity and its environment.	For example, the objective of a wrapped food or moving some food somewhere specific.
Material location change process	A material context change in which the objective is to move the input material to another location.	The ultimate location may be dynamically ascertained based on other inputs/decision points, for example, in a sorting process [this is also an Industrial Ontologies Foundry term (Kulvatunyou et al., 2022)].
Material context change by a mechanism	A material context change is when an action that changes the contextual relations of the input material entity is applied.	For example, pushing against an object may cause it to move.

**Figure 5:**
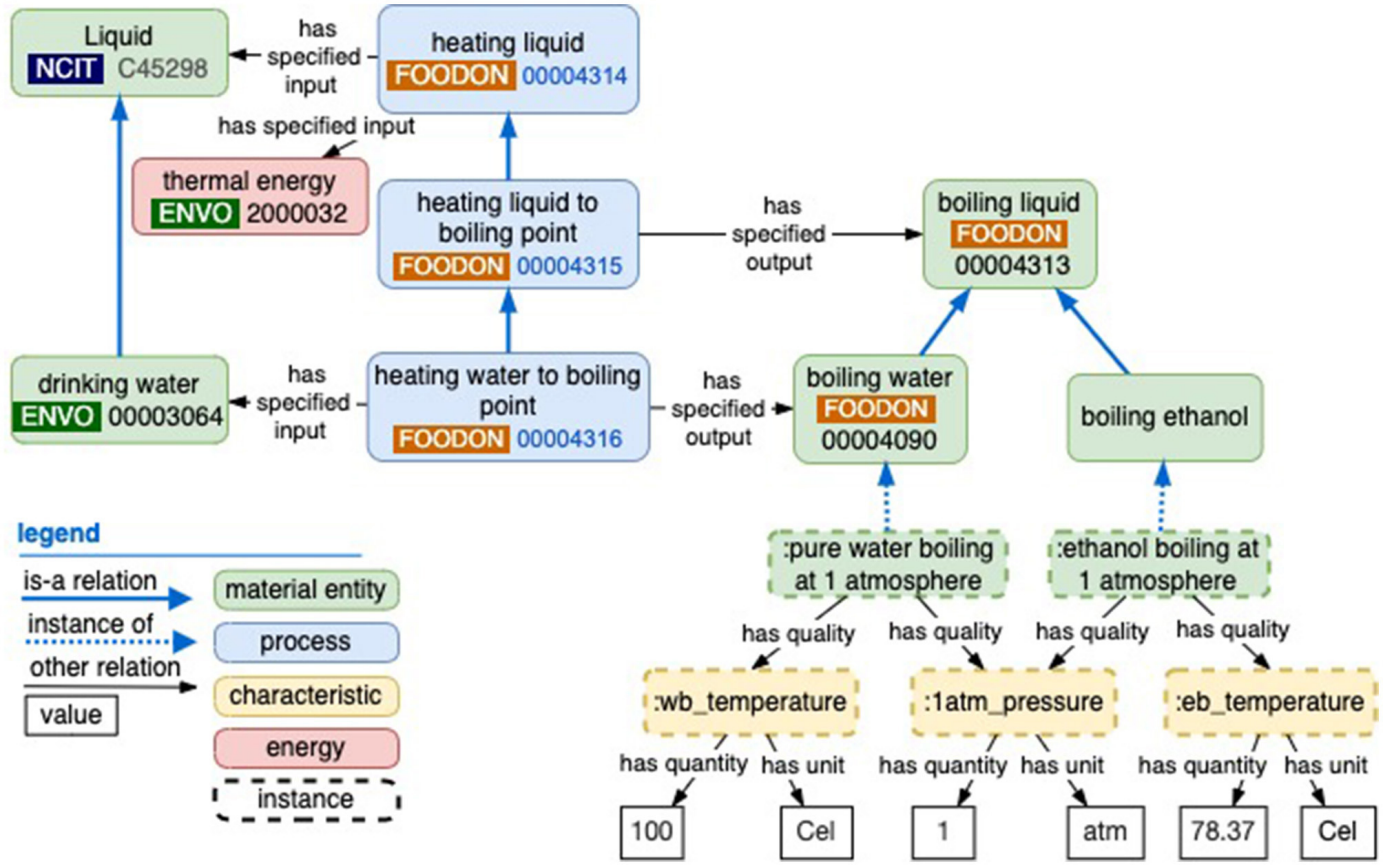
How an objective specification like “boiling water” can be expressed as a universal (class-level) output of a planned process. Boiling water is a class of boiling liquid with certain temperature and pressure characteristics, similar to other liquids like boiling ethanol.

The boiling water process example illustrates the distinction between processes which have more open-ended mechanisms, and those with completion objectives. A “heating liquid” class does not include any objective, but its “heating liquid to boiling point” subclass does require a boiling liquid output. More specifically, an objective to bring some potable or “drinking water” (usually at ambient temperature) to a boiling point may require some context for that boiling, e.g., the proxy objective of it being 100 degrees Celsius (°C) at 1 atmosphere (atm) unit. Additionally, a mechanism invoked to boil this water will require a liquid container, a vector of energy, and either a “heating of the container” process or a “heating by microwave” process. As shown in Figure 5, various liquids have different boiling point temperature x atmospheric pressure objectives. There is the potential for establishing a digital library of such instants—much like the SI library of real-world entities such as the meter and the kilogram—that can be reused to express process objectives. To model the process of “heating liquid to the boiling point,” one can reuse a URI that points to a “reference” instance of “boiling water” with its standard measurements of water and atmospheric conditions and a separate input instance of water with qualities that approach the standard over time (the multicomponent nature of these reference measures precludes a solution at the class level involving owl:hasValue; instead, the “has quantity” and “has unit” properties are in line with OBO's upcoming data model).

### 2.2. Molecular branch

The concepts of mechanism and objective apply to food at both macro and molecular levels, which gives rise to a correspondence between mechanism and objective activity at both levels. We describe the considerations for building the “molecular mechanism” branch and provide an example. This branch describes mechanisms specific to certain molecules that can be key to the molecular composition of a food's processed versions. There are at least two prominent characteristics of molecular-scale mechanisms: the chemistry of interactions between molecules and the spatial location of interactions within food material(s). Molecular interactions are either covalent (e.g., Maillard reaction) or non-covalent (including van der Waals forces, electrostatic forces, and hydrogen bonding) (Yamada, 2014) and may either occur throughout the material or be localized (Doi, 2013). Figure 8 provides a rice cooking example that identifies and differentiates various molecular mechanisms and sensor measurement concepts. Rice cooking is dominated by the molecular mechanism of starch interacting with water through different time and temperature conditions (starch comprises up to 90% of a rice kernel).

**Figure 6:**
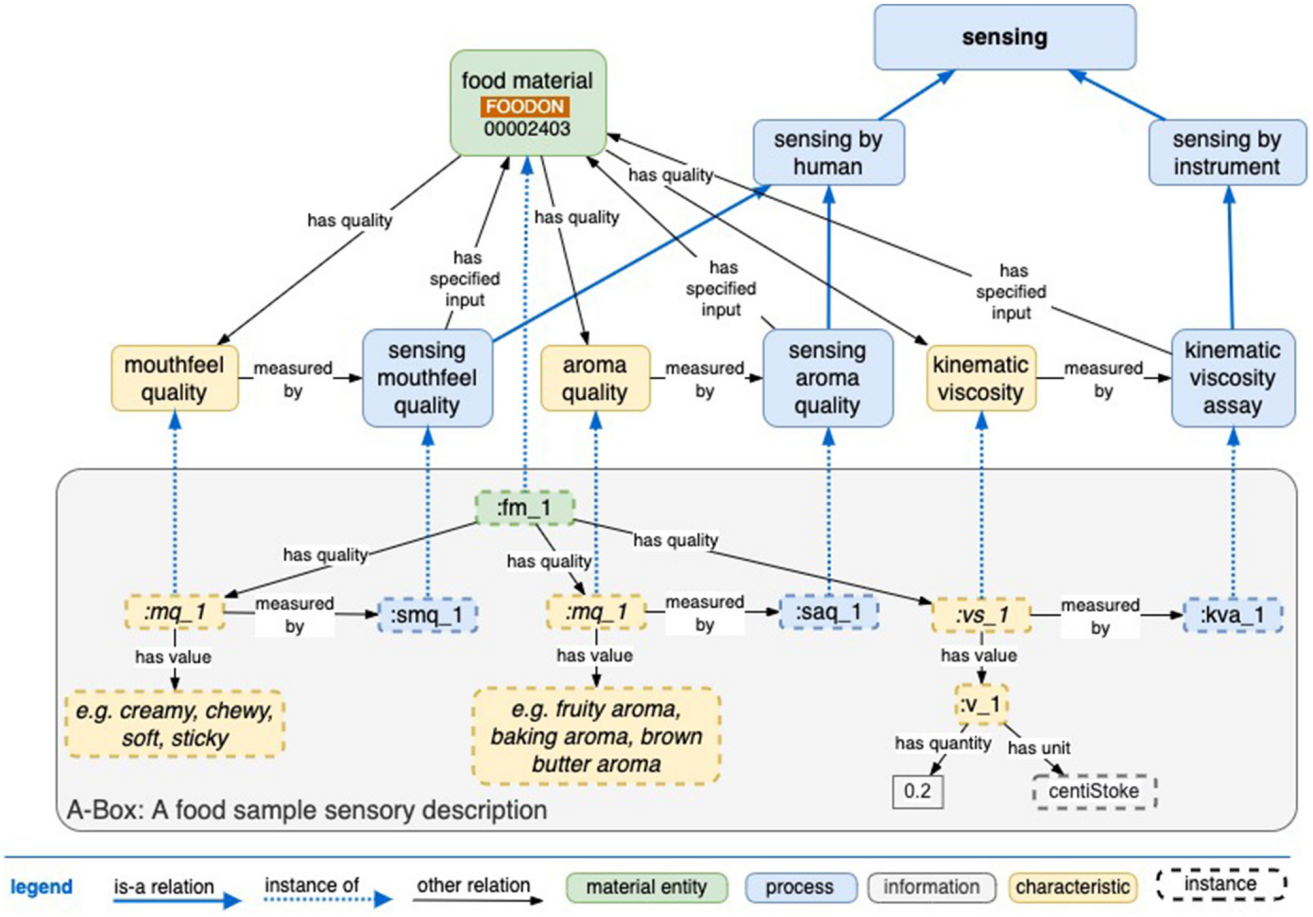
Generic schema of some sensory processes that help determine whether the objectives have been achieved during material processing.

The specific molecular processes are swelling, gelatinization, pasting, and retrogradation, all due to the hydrogen bonding interactions that occur in bulk when rice is cooked by soaking and boiling in water and then cooling. Initially, the components of starch, amylose (AM), and amylopectin (AP) are in the native “granular” state of alternating bands of amorphous and crystalline regions enabled by intramolecular hydrogen bonds. Soaking rice in water at ambient temperature sets off the gradual seepage of water into the structure at a rate proportional to the temperature. Heating this mixture of “soaked” rice and water increases the swelling of native starch granules. During this swelling process, the water creates hydrogen bonds with amylopectin and gradually disrupts the crystallinity of the granule irreversibly. This leads to the breaking of the native structure. Amylose and amylopectin leach into the water, and the gelatinization is complete. This is followed by pasting until the rice is “cooked.” AP and AM reassociate as the rice cools, a process termed retrogradation (Kadam et al., 2015). This is observed as the drying-out of the rice when refrigerated or the thickening of rice porridge (congee). These mechanisms can be sensed either by humans or an instrument and are associated with several objectives, as shown in Figure 6. Specific to the example explored above, instrumental sensors indicate rheological and physical properties, while humans sense the mouthfeel qualities described as stickiness, chewiness, creaminess, etc.

### 2.3. Applications

**Figure 7:**
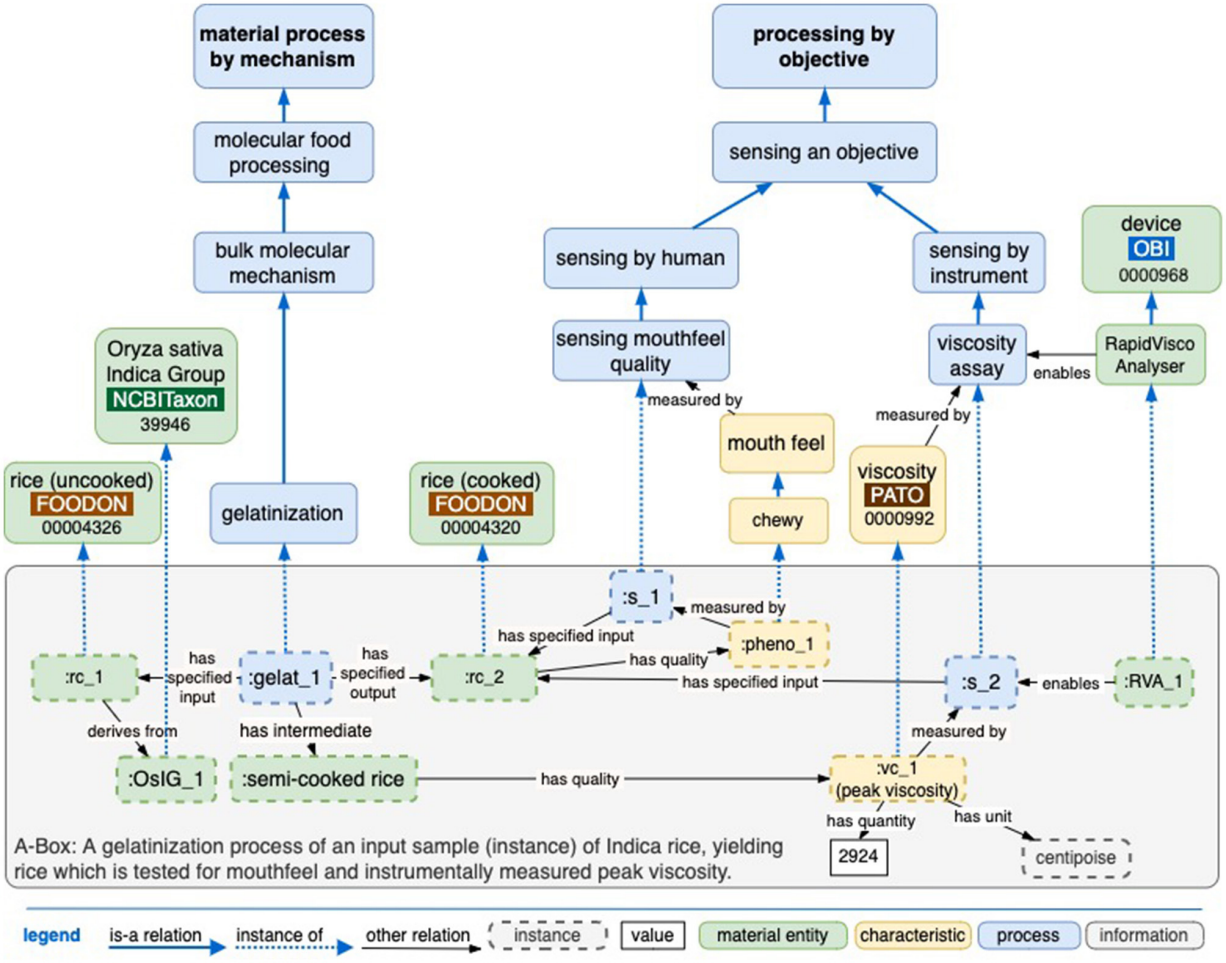
A basic model of rice cooking where completion is judged by some characteristic that is sensed by either humans or instruments. The A-Box (assertion box) expression uses the above T-Box (terminology box) ontology terms to express instances of experimental data.

The language and hierarchy of terms developed here apply to both scientific experiments and home cooking contexts, as shown in the context of the rice cooking example (Figure 7). From a domestic consumer-end recipe perspective, rice cooking often has a more formulaic approach, specifying a device and ingredient quantity, and completion is assumed by either the cooking time or by a sensory perception of mouthfeel (Naravane and Lange, 2018). From a food science perspective, rice cooking is described by the molecular mechanisms of swelling and gelatinization that specific instruments and protocols can measure. In addition, the language also addresses both macro-level and molecular-scale mechanisms, with the aim that changes in food composition can be explained at the molecular level.

The vast body of research literature on food processing addresses diverse questions to discover the sensory and nutritional profiles of processed foods due to processing conditions. Several experimental studies also aim to correlate objective measures with more subjective human sensory scales (Tao et al., 2020). Every experimental dataset typically explores only a few variables for specific outcomes but is taken together. These studies contribute to a vast body of research on food composition and transformative mechanisms. Integrating this experimental body requires a standardized language like this, and such large datasets have the potential for knowledge modeling, as evidenced by the models developed on traditional nutrition-focused datasets (Naravane and Tagkopoulos, 2023).

**Figure 8:**
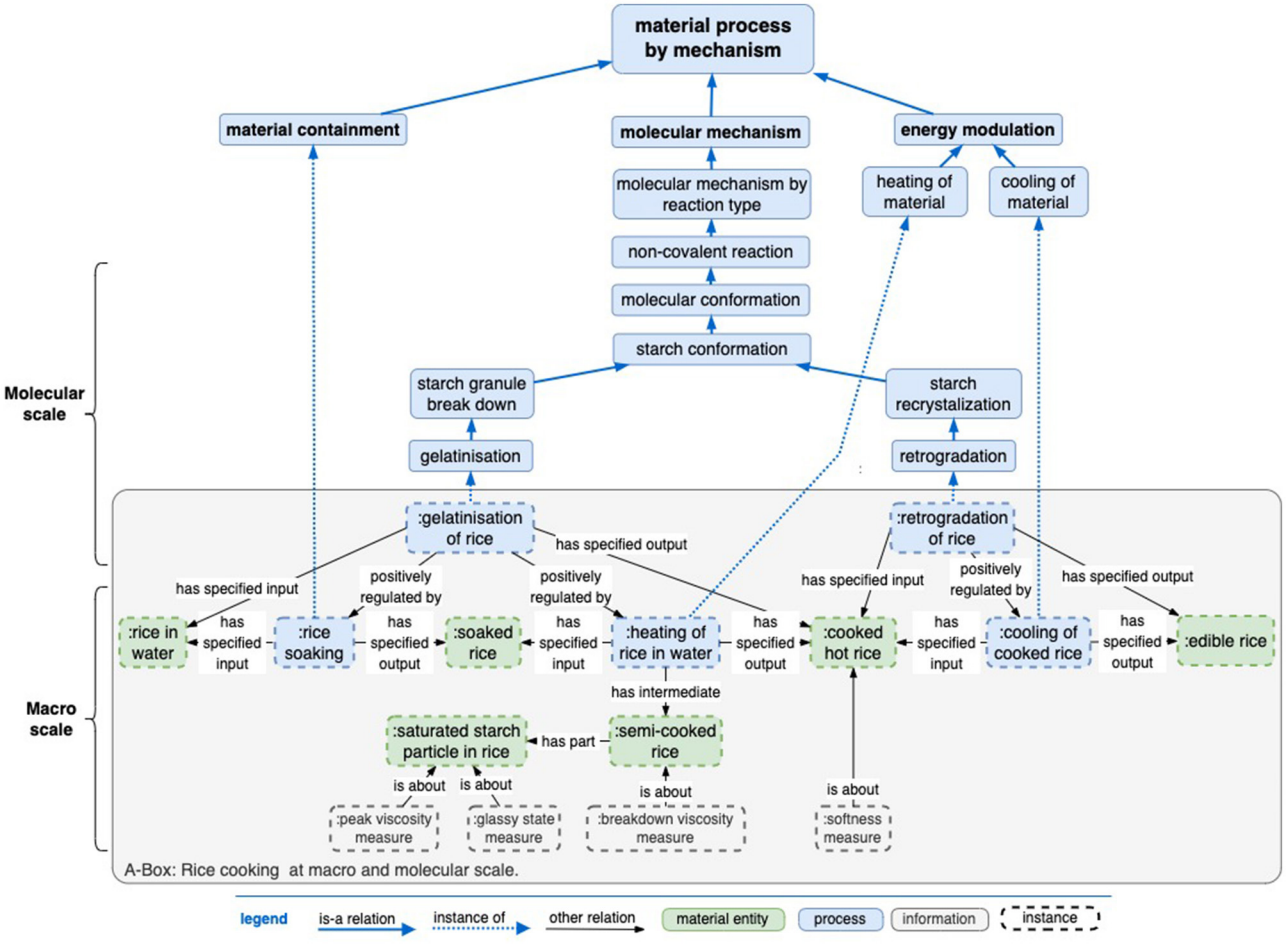
An example of a rice cooking process model can be viewed from both a macro and molecular scale, with an RO “positively regulated by” object property tying the two together.

Figure 8 illustrates the rice cooking use case of applying ontology to experimental studies. Rice cooking predominantly involves the interaction of water with rice through the energy supplied in the form of heat up to the boiling point of 100°C. A progression of rice states is shown in the material entity tier of the figure. Intermediate and final process products can be measured for experimental or process control variables. This abbreviated protocol omits some steps and controls one might have, such as washing rice, using a certain cooking device, setting the cooking temperature, etc. Specifically, the “gelatinization” process has been detailed since it is essential to cooking by virtue of the water penetrating the rice's native starch crystal structure, which advances to some extent in “soaking rice”, and the subsequent breakdown of crystal structure requires “heating of rice in water”. However, it will take more detailed modeling to address dried rice types such as having a pertinent kind of starch crystal formation, having husks removed, and water temperature factors to replace this simplified protocol.

Various material entities are observable in both “domestic” cooking and scientific experiments, while instrumental measures (such as peak viscosity and glass transition) that capture certain molecular states are specific to a scientific context. An example of dynamic process control involves modifying time and temperature conditions to affect two outcomes: the recrystallization of rice (which is associated with glycemic index) and the control of the textural properties of cooked rice (for example, soft, hard, and chewy).

Generally, ontology provides a formal language and framework to align various mechanisms, objectives, and instrumental measures. While the knowledge graph in this figure has been manually curated with food science expertise, this preliminary work could be evolved to support inference. Food science process terms such as peak viscosity and glass transition could also be text-mined from the literature and introduced under the ontology's mechanism and objective hierarchies at either a macro or a molecular scale.

The extracted terms can be used to structure data across food science experiments, and the analytical measurements associated with the process terms can be used for dynamic process control. Once finalized into ontologies such as OBI or FoodOn, this work will support data curation objectives—FAIR guidelines I1, R1.2, and R1.3 (FAIR Principles, 2017)—wherein data are coded in easily interpretable formats with precise provenance, and which use standardized (interoperable, reusable) language throughout. The chain of processes that ultimately generate data can be detailed as an instance of a protocol (whether experimental or operational), enabling a graph of a protocol's process, device, input, output, operator, and other contextual components—via ontology term and relation identifiers—to achieve disambiguation, comparability, and provenance of resulting datasets.

## 3. Future work and conclusion

This work should enhance clarity in finding a home for each food process under the matching mechanism/action or objective hierarchies. It should enable further research into how OWL logic can support the identification of equivalent processes for use in dynamic, versatile food processing pipelines. These elements are essential for enabling dynamic processing pipelines that can search and select from a library of processing components based on goal and/or resource constraints such as available tools or operators (mechanisms/actions) or material resources—a capability that humans often demonstrate in laboratory, industrial, or home food preparation settings. Additionally, this work should encourage the development of a better food processing protocol detail vocabulary, allowing appropriate comparison of data points within food composition databases and nutritional studies.

## Author contributions

All authors listed have made a substantial, direct, and intellectual contribution to the work and approved it for publication.

## References

[B1] Bandrowski A. Brinkman R. Brochhausen M. Brush M. H. Bug B. Chibucos M. C.. (2016). The Ontology for Biomedical Investigations. PLoS ONE. 11, e0154556. 10.1371/journal.pone.0154556 27128319 PMC4851331

[B2] Core Ontology for Biology Biomedicine (n.d.). Available online at: https://obofoundry.org/COB/ (accessed October 15 2022).

[B3] Doi M. (2013). “Surfaces and surfactants,” in Soft Matter Physics. Oxford: Oxford University Press. p. 51–71. 10.1093/acprof:oso/9780199652952.003.0004

[B4] Dooley D. Weber M. Ibanescu L. Lange M. Chan L. Soldatova L.. (2022). Food process ontology requirements. Semant. Web. (2022) 22, 1–32. 10.3233/SW-223096

[B5] Dooley D. M. Griffiths E. J. Gosal G. S. Buttigieg P. L. Hoehndorf R. Lange M. C.. (2018). FoodOn: a harmonized food ontology to increase global food traceability, quality control and data integration, NPJ Sci. Food. 2, 23. 10.1038/s41538-018-0032-6 31304272 PMC6550238

[B6] FAIR Principles (2017). GO FAIR. Available online at: https://www.go-fair.org/fair-principles/ (accessed November 3, 2022).

[B7] FoodOn Robot Tables. (2023). Google Docs. Available online at: https://docs.google.com/spreadsheets/d/1VJtz4m67tdUNDqRe3m1Okdxll64nTR46GSvCOmb0APE/edit (accessed October 15, 2022).

[B8] Hinojosa-Nogueira D. Pérez-Burillo S. Navajas-Porras B. Ortiz-Viso B. de la Cueva D. P. Lauria F.. (2021). Development of an unified food composition database for the European project “Stance4Health,” *Nutrients*. 13, 4206. 10.3390/nu13124206 34959759 PMC8704708

[B9] Jackson R. C. Matentzoglu N. Overton J. A. Vita R. Balhoff J. P. Buttigieg P. L.. (2021). BO Foundry in 2021: operationalizing open data principles to evaluate ontologies, BioRxiv. 06, 446587. 10.1093/database/baab069 34697637 PMC8546234

[B10] Kadam S. U. Tiwari B. K. O'Donnell C. P. (2015). “Improved thermal processing for food texture modification,” in Modifying Food Texture, Chen, J., and Rosenthal, A. (eds.). Sawston: Woodhead Publishing. p. 115–131. 10.1016/B978-1-78242-333-1.00006-1

[B11] Kulvatunyou B. Drobnjakovic M. Ameri F. Will C. Smith B. (2022). The Industrial Ontologies Foundry (IOF) Core Ontology. Tarbes: Formal Ontologies Meet Industry (FOMI). Available online at: https://tsapps.nist.gov/publication/get_pdf.cfm?pub_id=935068 (accessed June 25, 2023).

[B12] Naravane T. Lange M. (2018). Ontological Framework for Representation of Tractable Flavor: Food Phenotype, Sensation, Perception. Available online at: http://ceur-ws.org/Vol-2285/ICBO_2018_paper_45.pdf (accessed October 16, 2022).

[B13] Naravane T. Tagkopoulos I. (2023). Machine learning models to predict micronutrient profile in food after processing. Curr. Res. Food. Sci. 6, 100500. 10.1016/j.crfs.2023.100500 37151381 PMC10160345

[B14] Osorio S. Scossa F. Fernie A. R. (2013). Molecular regulation of fruit ripening. Front. Plant Sci. 4, 198. 10.3389/fpls.2013.00198 23785378 PMC3682129

[B15] Tao K. Yu W. Prakash S. Gilbert R. G. (2020). Investigating cooked rice textural properties by instrumental measurements, *Food Sci. Human Wellness*. 9, 130–135. 10.1016/j.fshw.2020.02.001

[B16] Temperatures Chilling Freezing Procedures. (2023). LII/Legal Information Institute. Available online at: https://www.law.cornell.edu/cfr/text/9/381.66 (accessed October 15, 2022).

[B17] Westenbrink S. Presser K. Roe M. Ireland J. Finglas P. (2021). Documentation of aggregated/compiled values in food composition databases; EuroFIR default to improve harmonization, J. Food Compost. Anal. 101, 103968. 10.1016/j.jfca.2021.103968

[B18] Yamada S. (2014). “Molecular interactions (molecular and surface forces),” in Encyclopedia of Polymeric Nanomaterials, eds S. Kobayashi and K. Müllen (Berlin; Heidelberg: Springer Berlin Heidelberg), 1–7.

